# Apple Fruit Diameter and Length Estimation by Using the Thermal and Sunshine Hours Approach and Its Application to the Digital Orchard Management Information System

**DOI:** 10.1371/journal.pone.0120124

**Published:** 2015-04-01

**Authors:** Ming Li, Meixiang Chen, Yong Zhang, Chunxia Fu, Bin Xing, Wenyong Li, Jianping Qian, Sha Li, Hui Wang, Xiaodan Fan, Yujing Yan, Yan’an Wang, Xinting Yang

**Affiliations:** 1 National Engineering Research Center for Information Technology in Agriculture, Beijing Academy of Agriculture and Forestry Science, Beijing, PR China; 2 State Key Laboratory of Crop Biology/College of Life Science, Shandong Agricultural University, Tai’an, Shandong, PR China; Zhejiang University, CHINA

## Abstract

In apple cultivation, simulation models may be used to monitor fruit size during the growth and development process to predict production levels and to optimize fruit quality. Here, Fuji apples cultivated in spindle-type systems were used as the model crop. Apple size was measured during the growing period at an interval of about 20 days after full bloom, with three weather stations being used to collect orchard temperature and solar radiation data at different sites. Furthermore, a 2-year dataset (2011 and 2012) of apple fruit size measurements were integrated according to the weather station deployment sites, in addition to the top two most important environment factors, thermal and sunshine hours, into the model. The apple fruit diameter and length were simulated using physiological development time (PDT), an indicator that combines important environment factors, such as temperature and photoperiod, as the driving variable. Compared to the model of calendar-based development time (CDT), an indicator counting the days that elapse after full bloom, we confirmed that the PDT model improved the estimation accuracy to within 0.2 cm for fruit diameter and 0.1 cm for fruit length in independent years using a similar data collection method in 2013. The PDT model was implemented to realize a web-based management information system for a digital orchard, and the digital system had been applied in Shandong Province, China since 2013. This system may be used to compute the dynamic curve of apple fruit size based on data obtained from a nearby weather station. This system may provide an important decision support for farmers using the website and short message service to optimize crop production and, hence, economic benefit.

## Introduction

Apples are one of the four most popular fruits worldwide because of their high fruit quality, which is important for human health [1]. Apple trees are perennial crops that have a production life of several 10s of years, requiring high levels of grower expertise and cultural management. In the Internet era, where information plays a key role in our life, fruit cultivation is rapidly becoming a very data intensive industry. For example, farmers make decisions based on huge volumes of information obtained from a diverse number of devices, such as meteorological sensors, farming machinery, and short messages, to enhance production and to ensure fruit quantity and quality for maximum economic gain [2]. As a result, farmers require model-based decision support systems to provide information services. Modeling systems were first used to enhance crop production in the mid-1970s, using mainframe computers [3]. Decision support systems in agriculture first appeared in the late 1980s and early 1990s, when desktop computers became more readily available and affordable; however, their application was limited to the time required to input data and retrieve useful information from the computers [4]. Fortunately, with the maturation of mobile technology, such as cellular phones and Personal Digital Assistants (PDAs), and the widespread adoption of the Internet, farmers are now able to collect agricultural production data and obtain decision-support by wireless devices wherever and whenever they want [5]. Digital agriculture, or the Internet of Things (IOT) in agriculture, has become increasingly popular; however, existing and future systems tend to operate under specific models that have a theoretical basis and scientific support [6]. In addition, model implementation is dependent on the decision support system being used [7]. Much work has been devoted to modeling the growth and development of fruit trees in relation to orchard management and decision-making processes [8]. In these models [9], trees react to their environment and management interventions by adjusting their physiological functions and structure [10]. However, experimental data based on optimized simulations are essential to ensure that models reflect fruit development on real-life trees [11].

The fruit shape index (FSI) is the ratio between fruit length and diameter, and represents one of the most important traits of apple fruit external quality. In East Asia, cultivars with large FSI have greater economic potential in the fresh-product market [12]. Many models of apple fruit size exist [13,14], but most focus on yield prediction [15]. Li et al. [16] established mathematical models of length and diameter using the logarithmic curve. Kaack and Pedersen [17] determined the relationship between fruit size, fruit weight, and climatic factors, and how these factors interact, at harvest to predict the optimal harvest date. Stajnko et al. [18] investigated the modified Gompertz function (models), which described apple fruit growth on different trees and the position of shoots on the trees. Most of these models are driven by developmental days calculated using the calendar, which is called calendar-based development time (CDT). CDT models have been developed over several hundred years, by counting the days that elapse between the beginning date (e.g., after full bloom) and the date on which the phenological event occurs (e.g., the fruits ripen) [19]. Most models use developmental days as the driving variable; however, fruit growth was also strongly influenced by weather conditions as well, causing a decrease in CDT model accuracy when incorporating data from different years [20].

Physiological development time (PDT) was proposed in 1997 [21], which differs to CDT because it combines important environmental factors, such as thermal and sunshine hours, into the models used to calculate the time required for crops to complete a given physiological stage. The accuracy of PDT models has been evaluated for field crop [22–25] and vegetable crop [26,27] datasets of different genotypes and latitudes [21]; however, reports on the effectiveness of this system for apple trees remain limited [28], especially with respect to apple fruit size estimations.

Thus, this study developed a simulation model of apple fruit size using PDT as the driving variable. We compared the results against the CDT model, and validated the model from field data. Finally, the model was implemented in a digital orchard management system conducted on web sites and cell phones.

## Materials and Methods

### Experimental orchard

This study was conducted from 2011 to 2013 in a private fruit orchard (latitude 36°14ʹN; longitude 116°50ʹE) at Chaoquan Town, Feicheng County, Tai’an, Shandong Province, China, with permission being provided by the host, Mr. Chengjun Yin. 12-year-old Fuji apple (*Malus domestica* Borkh.) trees were selected on seedling rootstock tea crabapple (*M*. *hupehensis* [Pamp.] Rehd.) trees of uniform canopy size (3.5 × 3 m) with a thinned spindle-type training system, so that the crop loads of all trees were similar: 235 ± 20 fruits per tree in 2011, 206 ± 16 fruits per tree in 2012, and 220 ± 15 fruits per tree in 2013. This area of China is frequently subjected to periods of high summer and warm autumn temperatures, with a moderate amount of rainfall (680 mm/year) in the last 30 years (from 1981 to 2010). The annual mean temperature was 15.6 ºC, 16.1 ºC, and 16.5 ºC in 2011, 2012, and 2013, respectively, at the site, with a rising trend being documented over the last 30 years (The annual mean temperature in 1984 was 12.3ºC). These records support the global warming trend [29, 30].

### Orchard environment monitoring

Three different rows of apple trees at random were selected, and the temperature, solar radiation, and soil moisture measured in each row using three orchard environmental monitoring devices (National Engineering Research Center for Information Technology in Agriculture, Beijing, China) at one-hour intervals. The three devices were placed between two apple trees in each row. The data were sent to a server in the office of the authors using general packet radio service (GPRS), and analyzed remotely.

### Fruit size measurement

Two apple trees located close to each environment monitoring device were chosen, and randomly marked 10 apples in the mid-upper part of shoots positioned at four compass directions, East, South, West, and North, in each tree. At an interval of about 20 (from 10 to 40) days after full bloom, fruit length and size were taken using a Vernier caliper until the fruit had ripened and, afterward it harvested. The mean values for apple fruit sizes on the trees near to each device on each sampling date were computed, with this information being used as a reference for the simulation model. The apple fruits were measured eight times in 2011 and five times in 2012 and 2013, so the datasets consisted of the means of 20 fruits per monitoring location at five to eight dates during each season. Twenty-four, fifteen and fifteen samples were collected in 2011, 2012, and 2013, respectively. The data in 2011 and 2012 were used to establish the models, while the data in 2013 were used to validate the models.

### Model development for fruit size simulation

Fruit development was computed after full bloom, and the driving variable was changed from time t to PDT. PDT is the sum of the relative physiological development effectiveness (RPDE), which is the combination of the relative thermal effectiveness (RTE) and relative sunshine-hour effectiveness (RSE).

The relationship between relative thermal effectiveness *RTE(T)* and temperature maybe described by the following Eq (1):

$$RTE(T)=\left\{\begin{array}{cc} 0 & \left(T<T_{b}\right)\\ \left(T-T_{b})/(T_{ob}-T_{b}\right) & \left(T_{b}\leq T\leq T_{ob}\right)\\ 1 & \left(T_{ob}\leq T\leq T_{ou}\right)\\ \left(T_{m}-T_{})/(T_{m}-T_{ou}\right) & \left(T_{ou}\leq T\leq T_{m}\right)\\ 0 & \left(T>T_{m}\right) \end{array}\right\}$$ (1)

where *RTE(T)* is the relative thermal effectiveness at daily temperature *T*, which is computed using mean hourly temperature; *T*_*b*_ is the lower temperature limit for apple development; *T*_*m*_ is the upper temperature limit for apple development; *T*_*ob*_ and *T*_*ou*_ are the lower and upper optimal temperature limits for apple development. These parameters were determined from the published literatures [31–33].

The relationship between relative sunshine-hour effectiveness *RSE(T)* and daily sunshine hours is described by the following Eq (2):

$$RSE(DL)=\{\begin{array}{cc} 0 & \left(DL\leq DL_{c}\right)\\ \left(DL-DL_{c})/(DL_{o}-DL_{c}\right) & \left(DL_{c}<DL\leq DL_{o}\right)\\ 1 & \left(DL>DL_{o}\right) \end{array}\}$$ (2)

where *RPE(DL)* is the relative photoperiodic effectiveness at the daily length of sunshine (*DL*); *DL* is defined as the number of daily sunshine hours (the time when solar radiation intensity ≥120 W/m^2^); *DLc* is the lower photoperiod limit for apple development; *DLo* is the optimum photoperiod for apple development [33].

RPDE=RTE×RSE (3)

PDT=sum(RPDE) (4)

where *PDT* is the sum of the *RPDE* each day after full bloom (*DAFB*) for apples.

CDT=sum(DAFB) (5)

where *CDT* is the sum of the *DAFB* of apples, which is calculated as an unit of days.

The relationship of PDT and CDT with fruit size was fitted following the logistic pattern using the nonlinear regression procedure (NLIN) with the Gauss–Newton method of the SAS program (Statistical Analysis System V.9.3, SAS Institute Inc., Cary, NC, USA).

The PDT model was expressed as:

Fruit diameter simulation: 8.3501/(1+8.4013*EXP(-0.0905*PDT)) (P < 0.0001) (6)

Fruit length simulation: 6.7741/(1+ 7.7702*EXP(-0.0944*PDT)) (P < 0.0001) (7)

In addition, we used the CDT model for reference and comparison purposes:

Fruit diameter simulation: 8.4394/(1+4.5501*EXP(-0.0245*CDT)) (P < 0.0001) (8)

Fruit length simulation: 7.1689/(1+ 2.8493*EXP(-0.0192*CDT)) (P < 0.0001) (9)

### Model evaluation method

The estimated and measured diameters of apple fruits were compared using random point figures. The graphical procedures in SAS were used to evaluate the model estimations facing actual measurements. Scatter plots of the model-estimated values were compared to the measured values were presented for each year. The REG Procedure of SAS was used to evaluate the linear relationship for the simulation versus the measurements for each year [34]. Statistical indicators were used to estimate errors in the model. The errors were evaluated by *RMSE*, Willmott agreement index (*W)*, mean absolute error (*MAE)*, and mean bias error (*MBE)* [35]; in which:

$$RMSE=\sqrt{\frac{\sum_{i=1}^{N}{\left(Pi-Mi\right)}^{2}}{N-1}}$$ (10)

$$W=1-\frac{\sum_{i=1}^{N}{\left(P_{i}-M_{i}\right)}^{2}}{\sum_{i=1}^{N}\left(|P_{i}^{'}|+|M_{i}^{'}|\right)}$$ (11)

$$MAE=\frac{\sum_{i=1}^{N}\left|P_{i}-M_{i}\right|}{N}$$ (12)

$$MBE=\frac{\sum_{i=1}^{N}\left(P_{i}-M_{i}\right)}{N}$$ (13)

where *P*_*i*_ is the estimated values, *M*_*i*_ is the measured values, *P′*_*i*_ is the deviations from mean estimated values, *M′*_*i*_ is the deviations from mean measured values, and *N* is the sample size.

### Model implementation

**Fig 1:**
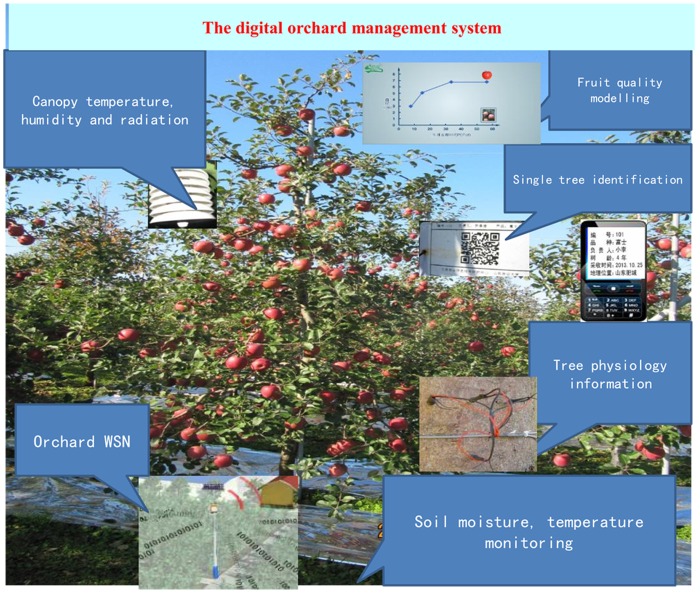
Model implementation in a digital orchard management information system.

The model was implemented by Visual Studio 2005 (Microsoft Corporation) to generate a web service for the management information system of a digital orchard (MISDO). The system had several practical functions, such as Viewing orchard information, Querying fruit tree information, Diagram of production record, and Statistical analysis. Using the data from a nearby weather database in the orchard, the mean diameter and length of apples could be estimated by the PDT model (Fig 1). Using this model, the system could determine the rapid growth period of apple fruits and recommend which treatment to apply, such as leaf fertilization and proper irrigation [1]. The established techniques were integrated into a digital orchard management and decision support system to provide information and recommendations for farmers using websites and short message services (SMS).

## Results

### Error analysis of fruit diameter response to the environment

**Fig 2:**
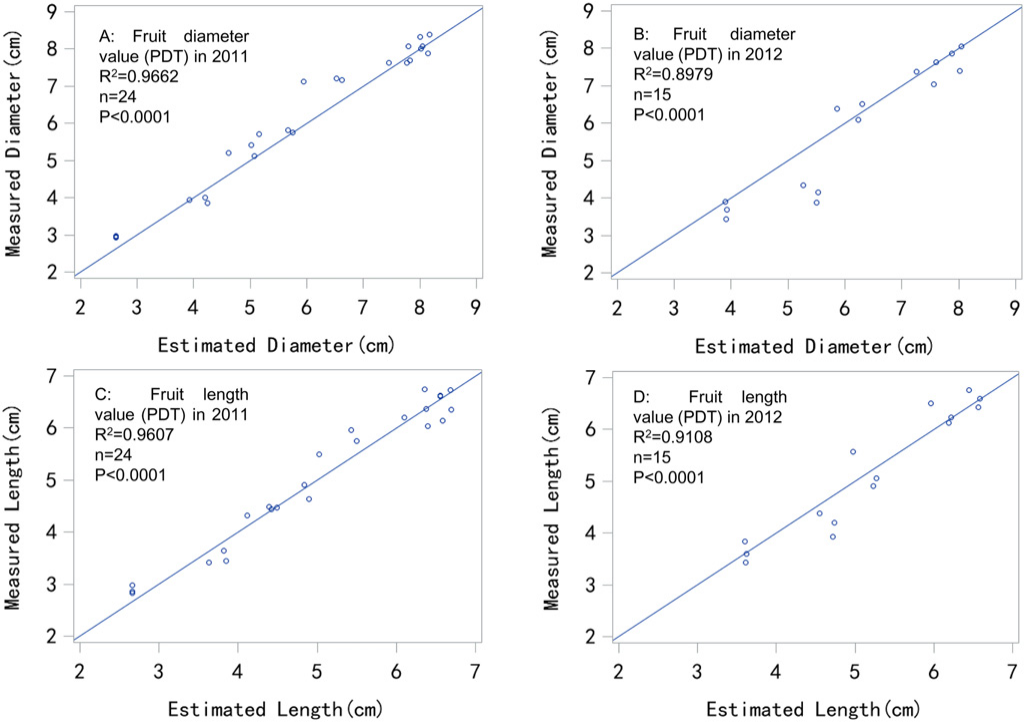
Comparison between estimated and measured fruit size values in 2011 and 2012 using the PDT model.

**Fig 3:**
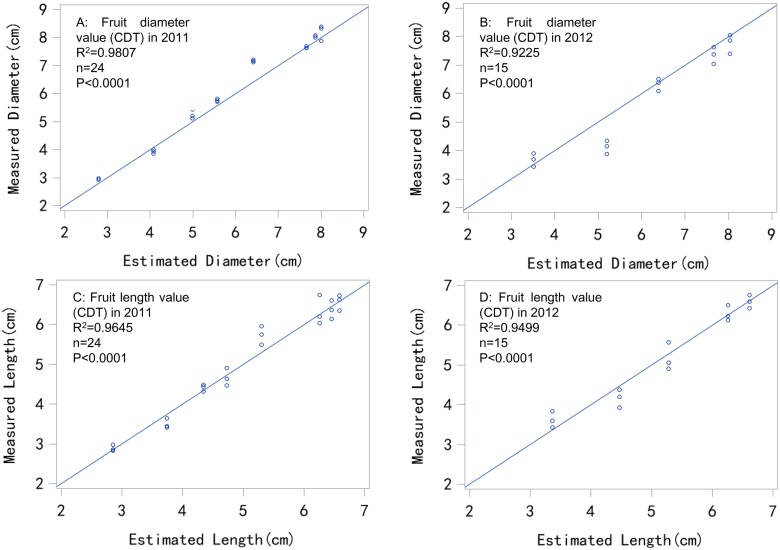
Comparison between estimated and measured fruit size values in 2011 and 2012 using the CDT model.

The measurement and simulation results using the PDT and CDT models in 2011 (Figs 2A and 3A) and 2012 (Figs 2B and 3B) were compared, and fitted well. The models were not significantly different based on the t-test. In the error analysis with the 2011 and 2012 results (Table 1), the PDT and CDT models performed similarly well, with accuracies of *R* ^*2*^ = 0.8881 to 0.9807 and *W* = 0.9263 to 0.9870. The estimation errors from 2011 and 2012 were less than 0.5 cm (*MAE* = 0.2494 to 0.4527). Thus, the two models could estimate fruit diameter to within 0.5 cm. The fruit size class at market is mostly expressed in millimeters, with the change from one class size to another generally being set at 5 mm or 0.5 cm intervals [36]. Therefore, this level of precision could help farmers meet the requirements for the apple fruit market.

**Table 1:** Error analysis of fruit diameter and length simulation in the spring and autumn of 2011, 2012, and 2013.

Item	Year	Model	*a* ^t^	*b* ^*u*^	*R* ^*2*^ ^*v*^	*W* ^*w*^	*MAE* ^*x*^	*MBE* ^*y*^	*RMSE* ^*z*^
Diameter	2011	PDT	0.9760	0.3550	0.9662	0.9796	0.3050	-0.2130	0.4119
		CDT	1.0211	0.0772	0.9807	0.9870	0.2494	-0.2019	0.3357
	2012	PDT	1.0803	-0.8316	0.8881	0.9263	0.4527	0.3347	0.6872
		CDT	0.9711	-0.1272	0.9225	0.9519	0.4017	0.3048	0.5795
	2013	PDT	0.8897	0.8112	0.9941	0.9916	0.1908	-0.1514	0.2791
		CDT	0.8869	0.5064	0.9600	0.9730	0.3634	-0.2109	0.4331
Length	2011	PDT	0.9669	0.1988	0.9607	0.9799	0.2182	-0.0326	0.2738
		CDT	1.0190	0.0692	0.9645	0.9816	0.1960	-0.0265	0.2571
	2012	PDT	1.0698	- 0.4121	0.9108	0.9506	0.2761	-0.0477	0.3732
		CDT	0.9617	0.1721	0.9499	0.9744	0.2213	-0.0272	0.2769
	2013	PDT	0.9429	0.4091	0.9830	0.9883	0.1488	-0.1194	0.2127
		CDT	0.9475	0.1395	0.9617	0.9768	0.2496	0.1406	0.2848

^t^ and ^u^: Regression coefficient determined by simple linear regression *Y = a+bX*, where estimated values = *X*, measured values = *Y*;

^v^: coefficient of determination;

^w^: Willmott agreement index;

^x^: Mean absolute error;

^y^: Mean bias error;

^Z^: Root mean square error.

**Fig 4:**
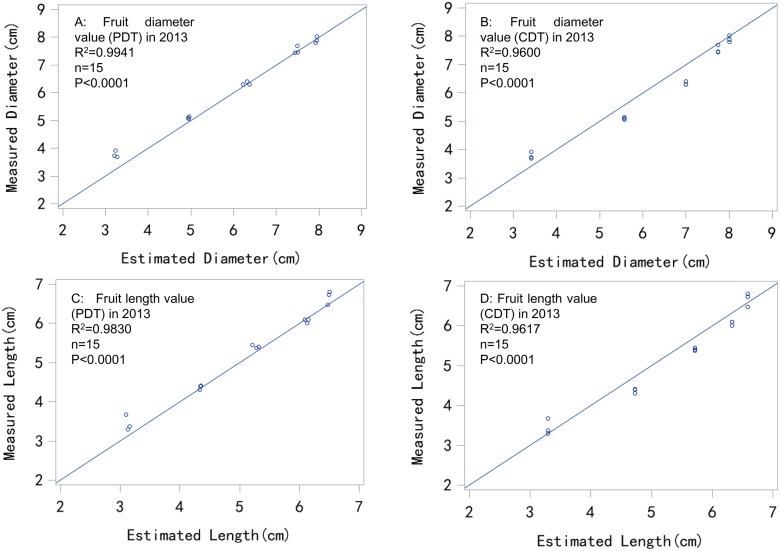
Comparison between estimated and measured fruit size values in 2013 using the PDT and CDT models.

Although the estimation results were similar for both the PDT and CDT models, the PDT model was more consistent than the CDT model, because the *MAE*, *MBE*, and *RMSE* of the PDT model were 0.1908, -0.1514, and 0.2791, respectively, in 2013 (Table 1, Fig 4A and 4B), which was better than that obtained for the CDT model. When extending the study to include a third year (2013), we found that the estimation error of the PDT model was rather stable (less than 0.2 for *MAE*), whereas that of the CDT model increased (to 0.3634 for *MAE*) (Table 1).

### Error analysis of fruit length response to the environment

The PDT and CDT models appeared to fit the fruit length measurement in 2011 (Figs 2C and 3C) and 2012 (Figs 2D and 3D) well, with similar results being obtained in the fruit diameter simulation. In the error analysis based on the 2011 and 2012 results (Table 1), both PDT and CDT models performed similarly well, with accuracies of *R* ^*2*^ = 0.9108 to 0.9645 and *W* = 0.9744 to 0.9816. The estimation errors in 2011 and 2012 were less than 0.5 cm (*MAE* = 0.1960 to 0.2761). Thus, the PDT model could provide length estimation with the error being within 0.3 cm, which was similar to the diameter estimation results.

Furthermore, the PDT model more consistently and accurately estimated fruit length than the CDT model, because the *MAE*, *MBE*, and *RMSE* of the PDT model were 0.1488, -0.1194, and 0.2127, respectively, in 2013. These parameters were 10.1%, 2.1%, and 7.2% lower than the CDT model (Table 1, Fig 4C and 4D).

### Operation examples of the system with model implementation

**Fig 5:**
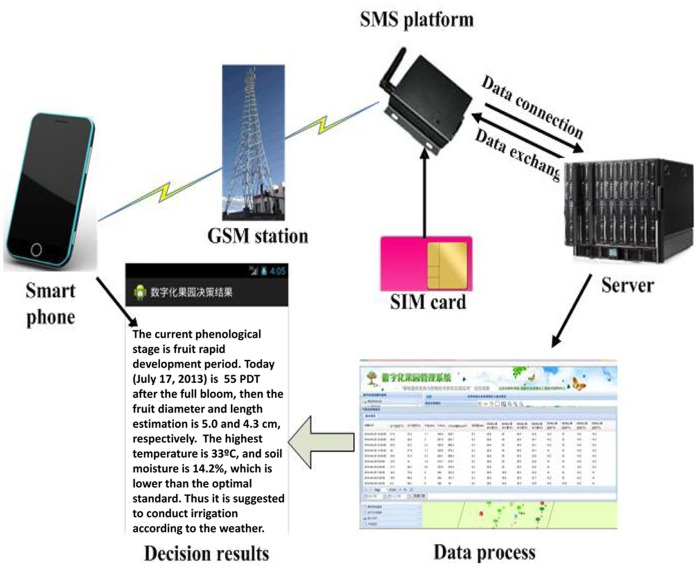
Schematic showing the use of digital orchard management information system.

The digital system had been applied in the same area of Shandong Province, China since 2013. The cases of application in which the system was applied are summarized in Table 2 based on users’ feedback, which supported the huge potential of the PDT models. Taking the irrigation decision support as an example, the system estimated the fruit diameter (5.0 cm) and length (4.3 cm) using the PDT model in July 17, 2013, which showed that apple fruit size increased more rapidly in July compared to the previous months, and that this stage represented the peak growth period for fruit size. Based on expert knowledge, the system made the suggestion to irrigate the trees soon because the apples were at a key developmental stage; specifically, air temperature had raised high recently, and the soil moisture sensor signaled a low water content. The suggestion was sent to the mobile phone of farmers using short message; thus, providing decision support services in sufficient time (Fig 5).

**Table 2:** The cases for application of the digital orchard management information system using the PDT model.

Name of use case	Description
Monitoring changes to fruit phenological stages	Farmers can use the system to estimate fruit size development and obtain real time data from remote places. It has huge potential for monitoring fruit growth without manual measurements.
Comparison with historical averages	By accumulating data over several years with this system, farmers may compare fruit growth of the current year to historical averages to identify specific trends, such as whether fruit diameter has decreased recently under similar management regimes in these years. Then farmers may adjust their management accordingly.
Tips for fertilization and irrigation at key stages	Based on real time data of fruit size development, we may determine the fertilization and irrigation date at key stages more precisely than previously.
Tips for improving the microclimate in orchards	The model could provide cultivation suggestions, such as proper training to improve light and ventilation conditions or alleviating the abnormal growth of apple fruit in summer due to high temperature.
Selecting the harvest date	Fruit size is an important indicator of external quality for determining harvest date. For Fuji apples, optimal fruit size is between 75 to 80 mm, which may be estimated using the PDT model.
Predicting production levels	The amount of apples produced depends on fruit volume and number or crop load. The model can estimate fruit diameter and length to help calculate fruit volume and quantity.
Estimating the fruit shape index	Fruit shape index (FSI) is one of the most important traits in apple fruit external quality, which is the ratio between the length and diameter of fruit. Real time data on fruit length and diameter may be used to estimate the fruit shape index.

## Discussion

The development of the PDT model for apple orchards is a major step forward in terms of improving the ability to estimate the fruit diameter and length. The independent year measurement data (i.e., 2013), validated the PDT model, showing that it performed better than the CDT model. The reason for this difference may be climatic differences during the production years, with the calendar-based estimation requiring accumulated data from many years, leading to inevitable errors. Similar results have been reported in previous research [17], with apple cultivation sites worldwide being frequently subjected to periods of high temperatures in summer and warm temperatures in autumn as well as varied sunshine hours [37]. Unlike the CDT mode, the PDT model was able to reflect the integrative effects of these variables. For example, the mean temperatures at the study site from April to October were 23.6 ºC, 23.9 ºC, and 24.3 ºC in 2011, 2012, and 2013, respectively. These temperatures indicate the likelihood of rapid fruit development, earlier fruit setting, and higher production levels. High temperature increases the speed at which fruit grows; thus, the PDT model uses the thermal unit to describe the temperature effects on fruit size development. Sunshine hours vary with latitude, and are essential for increasing fruit size [37], with fog, haze, and rain negatively affecting this variable. The number of sunshine hours has decreased annually since 1953 in the study area [29], with a similar trend being documented in other areas of China [38,39] and worldwide [40–42]. Fewer sunshine hours in April and August may affect photosynthesis and reduce the speed of fruit growth, in addition to decreasing ascorbic acid and sugar content in the fruit [41]. The PDT model uses relative sunshine-hour effectiveness (RSE) to explain the sunshine hour effects on fruit size. Thus, the PDT model could provide a better estimate of apple fruit size in different years.

Here, the MISDO was developed to link with sensors, models, and systems, facilitating the provision of short message services to farmers. The system overcame the barrier of data input for decision making [7,43], and operated under the proposed model predicating the mean fruit size in the orchard. Using the short message service, information about when and how to optimize fruit size using irrigation, fertilization, training, and microclimate control at the key stage of apple development were provided to farmers. Furthermore, fruit size is the basis of fruit weight, production, and external quality. Thus, our results could be used to estimate total production levels and external fruit quality, which is particularly important for ensuring the future size grade status of fruit with the help of the agricultural meteorological service. In future, a range of fruit physiological characteristics, including crop load, tree vigor, and source/sink relationships, water availability, rainfall, fruit temperature, and fruit transpiration [44], will be integrated into models predicting fruit growth, ripening, and quality development.

## Conclusions

PDT is the sum of the RPDE, and integrates thermal and sunshine hours, which are the top two most important environmental factors that influence apple fruit size. Unlike CDT, PDT is able to take into account variation in thermal and sunshine hours among years, facilitating greater flexibility in predictions. Using the measurements of fruit in an apple orchard during 2011 and 2012, four logistic models of fruit size (including diameter and length development) were computed using the PDT and CDT as the driving variables. Both models had similar performance based on the data used for model-establishment; however, the PDT model estimated the mean diameter and length of apple fruits with an error of within 0.2 cm in an independent year (2013), confirming that it was more robust than the CDT model. Furthermore, the PDT model provided the basis for predicting fruit weight, production, and external quality. The PDT model was also implemented into a digital orchard management information system to help farmers optimize fruit size by using fertigation, training, and microclimate control during the key stages of apple development.

## Supporting Information

S1 Table The measured data of apple fruit size. (XLS)
